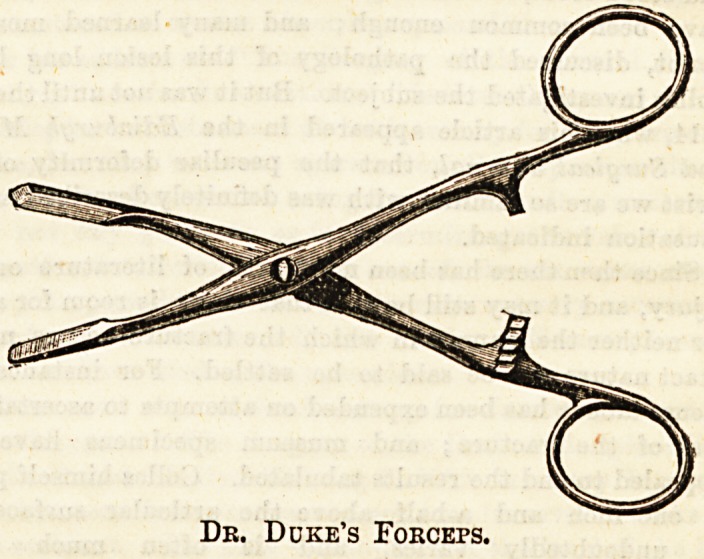# New Drugs, Appliances, and Things Medical

**Published:** 1892-05-28

**Authors:** 


### NEW DRUGS, APPLIANCES, AND THINGS MEDICAL.

[All preparations, appliances, novelties, etc., of which a notice is desired, should be sent for The Editor, to care of The Manager, 140, Strand, London, W.C.]

### INGROWING TOE-NAIL AND SOME MODERN INSTRUMENTS FOR ITS CURE.

There are several ways of treating this troublesome and common complaint. These may be divided into palliative and radical. First, then, let us look at the causes of the disease. Heister's "Surgery," 5th Ed., 1753, says, "The most general cause of this disorder is the wearing of too straight or narrow-toed shoes; which they will do well to avoid who are desirous of being free from the complaint." Erichsen says the same, and we see the complete explanation in the "Aseptic and Antiseptic Surgery" of Dr. Gerster, Ed. 1888, "Sweating feet, in combination with lack of cleanliness, improperly trimmed toe-nails, and narrow-toed shoes, offer the best conditions for the development of ulcerative processes near the anterior margin of the nail. Wherever the nail is trimmed off too short the adjacent skin will overlap its angle. The epidermis being macerated and soft from the profuse sweating, a small amount of friction between the edge of the nail and the skin will be sufficient to cause an excoriation. The pyogenic germs so abundantly present in the fœtid epidermal masses of sweating feet will not only come into contact with the raw surface, but will be rubbed into the open lymphatics by each successive step taken by the individual. An ulcerative inflammation of the parts results which offers poor conditions for natural drainage. Retention of the secretions leads to chronic suppuration, and to the extension of the process backwards towards the root of the nail, and also under it, until more or less of it becomes undermined and detached. Exuberant granulations, subject to frequent ulcerative destruction, spring up from the hypertrophied and infiltrated overlapping skin, and if unchecked the disorder terminates in the loss of the nail." He goes on to say that "the initial stages can often be successfully met with a careful local treatment. Curiously enough, his local treatment, which is antiseptic in its method, was anticipated by old Heister, who used, after washing and soaking, to place under the nail 'a compress dipped in warm spirit of wine,' which is a powerful germicide."

These palliative methods, however, only succeed in the milder cases. In fact, Erichsen goes so far as to say, "Various methods have been devised, with a view of raising the edge of the nail, partially removing and pressing aside the soft structures. I have never, however, seen much benefit result from any of these means." Here we might just mention a palliative method invented by Dr. Arthur Rowe, of Margate, and tried with success when he was House Surgeon to St. Mary's. A strip of thin silver about half to a-third of an inch broad is bent in the shape of the italic letter *S*; one limb rests under the edge of the offending nail, and the other over the back of the next toe, and is kept there by plaster. After a short time the steady leverage exerted drives up the depressed edge and compresses the swollen tissues by its side. If at the same time antiseptic dressings are kept applied, a cure is often effected.

Supposing, however, that milder means have failed to cure the disease, two methods yet remain. One, that which Dr. Gerster practices, is to cut off the exuberant tissues outside of the nail, and to cut out by knife and forceps a narrow piece of adjacent nail, taking care to destroy any shreds of the cut off matrix, the whole being performed antiseptically, and the dressing being an aseptic elastic one. The author has treated over a hundred of these cases in the manner described with the best results, the majority being patients of the German Dispensary, who walked to and from the institution during the time of treatment. The remaining and commonest form of treatment is that of evulsion of the whole nail, and here Erichsen states that it is the best and surest method. The way in which it is performed is as follows: A grooved director is thrust down to the quick in the middle of the nail, and a strong scalpel is then made to cut its way through the nail, or a pair of scissors may be used instead of director and scalpel. Then each half of the nail is twisted out of its bed by a tortion or other firmly-holding forceps. After trimming up the rough ends remaining, an aseptic dressing is applied, and the nail gradually grows again, and, by care, it is prevented from in-growing.

Lately Mr. A. G. Field, F.R.C.S., has mentioned a method which has yieldedg ood results in his hands. He says (*Lancet*, January 16th, 1892), "Observing, as all must have done, the readiness with which a splinter of wood inserts itself beneath the nail, I take an ordinary piece of deal about four inches long, pare one end thin, and adapt it to the size of the nail to be removed. The thin edge (previously oiled) is passed rapidly beneath the nail down to the root, and the hand raised, thus completely detaching it from its bed." Mr. Field has very courteously forwarded to us a specimen of his instrument. This particular one is fashioned out of well-seasoned elder, and is, in fact, an elder gouge.

Dr. Duke's Forceps.

Dr. Alexander Duke, of Dublin, has further worked on this idea, and has invented a forceps for the purpose. Messrs. Hockin, Wilson, and Co., London, the well-known purveyors for the medical profession, are making these. Through their kindness we show a sketch of them, and have also received a pair for inspection. Dr. Duke says: "The sharp-edged blade is intended to be introduced underneath the nail down to the root, the forceps then closed, the blades being held firmly together by the clip, the hand suddenly raised and the nail levered out of its bed. I fancy the forceps will be found a useful addition to every general hospital instrument cupboard, and will prove an improvement on the splitting and evulsion operation." We have but one word to add, and that is on the cutting of the nails. Cut them straight across and never by any means round the edges. The reason will be found in Dr. Gerster's description of the formation of an ingrowing toe nail. Last of all wear easy fitting boots and keep the feet clean and dry. The neglect of these common precautions will necessitate, notwithstanding an anæsthetic, what Heister states, viz., "an operation which will give the patient no small pain for a time," but he adds "yet he will quickly perceive the advantage by a more lasting ease."

## Figures and Tables

**Figure f1:**